# An isoform of 14‐3‐3 protein regulates transbilayer lipid movement at the plasma membrane

**DOI:** 10.1002/1873-3468.70338

**Published:** 2026-04-15

**Authors:** Akiko Yamaji‐Hasegawa, Akira Hattori, Masafumi Tsujimoto, Toshihide Kobayashi

**Affiliations:** ^1^ Lipid Biology Laboratory RIKEN Saitama Japan; ^2^ Laboratory for Neural Cell Dynamics, CBS, RIKEN Saitama Japan; ^3^ Department of System Chemotherapy and Molecular Sciences, Graduate School of Pharmaceutical Sciences Kyoto University Kyoto Japan; ^4^ Faculty of Pharmaceutical Sciences Teikyo‐Heisei University Tokyo Japan; ^5^ UMR 7021 CNRS University of Strasbourg Illkirch France; ^6^ Cellular informatics laboratory, RIKEN CPR Saitama Japan; ^7^ Holo‐Bio Co.Ltd Kyoto Japan; ^8^ Kyoto University of Advanced Sciences Kyoto Japan

**Keywords:** 14‐3‐3ζ, flip‐flop, fluorescent lipid analog, phosphatidylserine, transbilayer lipid movement

## Abstract

Exogenous phosphatidylserine (PS) is cytotoxic to Chinese hamster ovary (CHO) cells with a PS‐resistant CHO cell mutant exhibiting impaired transbilayer movement of a fluorescent PS analog at the plasma membrane. Here, we demonstrate that both mRNA and protein levels of 14‐3‐3 zeta are markedly reduced in this mutant, while knockdown of 14‐3‐3 zeta in wild‐type CHO cells confers partial resistance to exogenous PS. Consistently, these knockdown cells display defective transbilayer movement of 1‐palmitoyl‐2‐{6‐[(7‐nitro‐1,3‐benz‐2‐oxadiazol‐4‐yl) amino] hexanoyl}‐*sn*‐glycero‐3‐phosphoserine (C_6_‐NBD‐PS). However, 14‐3‐3 zeta knockdown does not further enhance PS resistance in ATP11C‐deficient cells. These findings indicate that 14‐3‐3 zeta plays a regulatory role in ATP11C‐dependent transbilayer movement of PS at the plasma membrane.

**Impact statement**

14‐3‐3ζ regulates ATP11C‐dependent phosphatidylserine (PS) transbilayer movement at the plasma membrane. Reduced 14‐3‐3ζ impairs PS flipping and confers resistance to exogenous PS in CHO cells, identifying 14‐3‐3ζ as a key modulator of phospholipid asymmetry and lipid‐induced cytotoxicity.

## Graphical abstract

Loss of 14‐3‐3ζ in CHO cells confers resistance to exogenous phosphatidylserine (PS) and impairs endocytosis‐independent inward flip‐flop of fluorescent PS at the plasma membrane. RNAi‐mediated knockdown reproduces this defect, while no additive effect is seen in ATP11C‐deficient cells. Thus, we establish 14‐3‐3ζ regulates transbilayer PS movement at the plasma membrane.

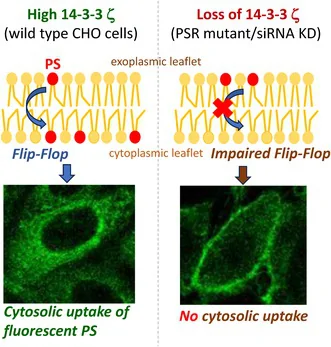

## Abbreviations

**C**_**6**_ **‐NBD‐PS**, 1‐palmitoyl‐2‐{6‐[(7‐nitro‐1,3‐benz‐2‐oxadiazol‐4‐yl) amino] hexanoyl}‐sn‐glycero‐3‐phosphoserine

**CHO**, Chinese hamster ovary

**P4‐ATPase**, type IV P‐type ATPase

**PS**, phosphatidylserine

The spontaneous transbilayer movement (flip‐flop) of phospholipid is intrinsically slow due to a high energy barrier. However, specific proteins facilitate this process [1, 2]. The translocation of phosphatidylserine (PS) from the exoplasmic to the cytosolic leaflet of the plasma membranes is catalyzed by type IV P‐type ATPases (P4‐ATPases). These are multispan transmembrane pumps that couple ATP hydrolysis to the active transport of lipid substrates against their concentration gradients. Most P4‐ATPases form heterodimers with the accessory subunit Cdc50—a partnership essential for their proper localization and activity.

In Chinese hamster ovary (CHO)‐K1 cells, the P4‐ATPase ATP11C plays a key role in PS flipping and has been implicated in the defective endocytosis‐independent cytosolic incorporation of 1‐oleoyl‐2‐{6‐[(7‐nitro‐1,3‐benz‐2‐oxadiazol‐4‐yl) amino] hexanoyl}‐*sn*‐glycero‐3‐phosphoserine in UPS‐1 (uptake of fluorescent PS analogs) mutant [3, 4].

We and others have previously reported that exogenous PS is cytotoxic to CHO cells [5, 6, 7]. From these studies, we isolated a PS‐resistant mutant, PSR (PhosphatidylSerine‐Resistant) 406. Similar to UPS‐1, PSR406 displays a defect in the endocytosis‐independent uptake of PS. In this study, we investigated the gene responsible for PS resistance and impaired PS uptake in PSR406 cells. Our results show that both mRNA and protein levels of 14‐3‐3 zeta (14‐3‐3ζ) are markedly reduced in PSR406 cells. Furthermore, RNAi‐mediated knockdown of 14‐3‐3ζ in wild‐type CHO cells conferred partial resistance to exogenous PS and a defect in the cytosolic incorporation of 1‐palmitoyl‐2‐{6‐[(7‐nitro‐1,3‐benz‐2‐oxadiazol‐4‐yl) amino] hexanoyl}‐*sn*‐glycero‐3‐phosphoserine (C_6_‐NBD‐PS). These findings suggest that 14‐3‐3ζ regulates the inward transbilayer movement of PS at the plasma membrane.

## Materials and methods

### Materials

L‐α‐phosphatidylserine (porcine brain; PS) and 1‐palmitoyl‐2‐{6‐[(7‐nitro‐1,3‐benz‐2‐oxadiazol‐4‐yl) amino] hexanoyl}‐*sn*‐glycero‐3‐phosphoserine (C_6_‐NBD‐PS) were purchased from Avanti Polar Lipids (Alabaster, AL, USA). Cell Count Reagent SF and Sepasol RNA I Super were from Nacalai Tesque (Kyoto, Japan). SuperScript III First‐Strand Synthesis System was from Invitrogen (Carlsbad, CA, USA). Thunderbird SYBR qPCR Mix was from TOYOBO (Osaka, Japan). ECL Prime Western Blotting Detection Reagents were from GE Healthcare (Buckinghamshire, UK). Rabbit polyclonal anti‐14‐3‐3ζ antibody (C‐16) was from Santa Cruz Biotechnology (Dallas, TX, USA). Mouse monoclonal anti‐β‐actin antibody (AC‐15) was from Sigma‐Aldrich (St. Louis, MO, USA). Horse radish peroxidase (HRP)‐conjugated anti‐mouse IgG and anti‐rabbit IgG were from Cytiva (Marlborough, MA, USA). Dynasore was from AdipoGen Life Sciences (Fuellinsdorf, Switzerland). siRNAs targeting 14‐3‐3ζ (1: GCCAUUGCCGAACUUGAUACC, 2: CGCUAAUAAUGCAGUUACUGA) and a control siRNA targeting GFP (GGCUACGUCCAGGAGCGCACC) were obtained from TaKaRa (Shiga, Japan). Lipofectamine RNAiMAX and Alexa Fluor 594‐conjugated dextran (10 000 MW) were purchased from Invitrogen.

### Cell culture

All cell lines used in this study were authenticated within the past 3 years. The CHO‐K1 cell line was a kind gift from Drs. M. Murata and S. Ohnishi (Kyoto University). The PS‐resistant mutant cell line (PSR406) was established as previously described [5], and the ATP11C‐deficient mutant cell line (UPS‐1) [3, 4] was kindly provided by Dr. Hanada (National Institute of Infectious Diseases and Japan Institute for Health Security). Authentication of the cell lines was based on their documented origin and the established derivation procedures for the mutant lines. Cell morphology and growth characteristics were consistent with the expected properties. Cells were maintained at low passage numbers after thawing from frozen stocks and were routinely tested for mycoplasma contamination. Cells were cultured in Ham's F‐12 medium supplemented with 10% fetal calf serum (FCS), 100 U/mL penicillin, and 100 μg/mL streptomycin at 37 °C in a humidified incubator with 5% CO₂.

### Cytosolic incorporation of C_6_ ‐NBD‐PS in living cells

Cells were cultured on glass‐bottomed dishes, washed three times with ice‐cold FCS‐free DMEM/Ham's F‐12 (1 : 1), and incubated with 3 μm C_6_‐NBD‐PS in FCS‐free DMEM/Ham's F‐12 at 6–7 °C for 5 min. After washing with ice‐cold medium, cells were either immediately observed or incubated at 37 °C for 15 min before observation. To remove C_6_‐NBD‐PS remaining at the cell surface, cells were incubated twice in Ham's F‐12 containing 10% FCS at 6 °C for 10 min and then washed with ice‐cold DMEM/Ham's F‐12 prior to imaging.

Images were acquired using an LSM510 confocal microscope (Carl Zeiss, Oberkochen, Germany) equipped with a C‐Apochromat 63×/1.2 NA water‐immersion objective or a TCS SP8 confocal microscope (Leica, Wetzlar, Germany) equipped with a 63× HC PL APO CS2 oil immersion objective. Fluorescent intensity was quantified using ImageJ software.

### Effect of dynasore on the endocytosis of Alexa Fluor™ 594‐ dextran in living cells

CHO‐K1 cells were cultured on glass‐bottomed dishes, washed twice with FCS‐free DMEM/Ham's F‐12 medium, and incubated with 0.5 mg/mL Alexa Fluor 594 ‐dextran at 37 °C for 15 min. After washing twice with FCS‐free DMEM/Ham' F‐12 medium, cells were observed using a TCS SP8 confocal microscope as described above.

To inhibit endocytosis, cells were pretreated with FCS‐free medium containing 100 μm dynasore for 10 min, followed by incubation with Alexa Fluor 594‐dextran in the presence of dynasore at 37 °C for 15 min. 100 μm dynasore was included in the washing medium throughout the washing and imaging procedures.

### Screening of differentially expressed genes between CHO‐K1 and PSR406 cells

Total RNA from CHO‐K1 and PSR406 cells was extracted with Isogen reagent (NIPPON GENE, Tokyo, Japan). Poly(A) + mRNA was purified from 400 μg of total RNA using the μMACS mRNA Isolation Kit (Miltenyi Biotec GmbH, Bergisch Gladbach, Germany). Differential gene expression analysis was performed using the PCR‐Select cDNA Subtraction Kit (Clontech Laboratories, Palo Alto, CA, USA) according to the manufacturer's protocol. Briefly, 2 μg of mRNA from each cell line was reverse‐transcribed into single‐stranded cDNA using a cDNA synthesis primer (42 °C, 1.5 h), followed by synthesis of double‐stranded cDNA (16 °C, 30 min). The cDNA was digested with Rsa I (37 °C, 1.5 h), and two adaptors (Adaptor 1 and Adaptor 2R) were ligated to the blunt‐ended fragments using T4 DNA ligase (16 °C, 16 h).

To enrich for genes specifically expressed in CHO‐K1 cells, adaptor‐ligated CHO‐K1 cDNA was hybridized with excess RsaI‐digested PSR406 cDNA (without adaptors) in hybridization buffer at 68 °C for 8 h. The two hybridization samples were then mixed and further incubated at 68 °C for 12 h. Specific PCR amplification of CHO‐K1 genes was performed using primers complementary to the 3′ ends of Adaptor 1 and Adaptor 2R with Advantage PCR Polymerase (Clontech). A parallel procedure was used to identify PSR406‐specific genes. The CHO‐K1‐specific PCR products were cloned into the pGEM‐T Easy vector (Promega, Madison, WI, USA) and transformed into *E. coli* ElectroMAX DH10B cells (Invitrogen). Plasmid DNA was prepared from 384 independent clones using the QIAGEN Miniprep Kit (QIAGEN, Hilden, Germany).

Plasmids were denatured and spotted onto Biodyne Plus positively charged nylon membrane (Pall, Port Washington, NY, USA) using a dot blot apparatus (Schleicher & Schuell, Keene, NH, USA). The membranes were hybridized with ^32^P‐labeled CHO‐K1 or PSR406 subtracted cDNA probes and analyzed using a BAS‐3000 image analyzer (Fuji, Kanagawa, Japan). DNA inserts from 129 plasmids showing specific hybridization signals were sequenced using a Taq dye terminator cycle sequencing kit and an ABI 377 DNA sequencer (Applied Biosystems, Foster City, CA, USA). Gene expression levels in CHO‐K1 and PSR406 cells were confirmed by virtual Northern blot analysis using subtracted cDNA and ^32^P‐labeled probes.

### Reverse transcription‐polymerase chain reaction (RT‐PCR)

RT‐PCR was performed as described previously [8].

### RNA interference

Small interfering RNA (siRNA) transfections were performed using Lipofectamine RNAiMAX according to the manufacturer's instructions. Cells were seeded in 6‐well plates and transfected with 25 pmol siRNA per well for 24 h. Subsequently, cells were harvested and reseeded into either 48‐well plates for cell viability assays or 6‐well plates for immunoblot analysis. Cells were cultured for an additional 24 h in the continued presence of siRNA prior to downstream experiments.

### Cell fractionation

For separation of membrane and cytosolic fractions, cells were washed three times with ice‐cold PBS and harvested by scraping in homogenization buffer (3 mm imidazole, pH 7.4, 8.5% sucrose). Cell suspensions were centrifuged at 190 × ***g*** for 5 min at 4 °C, followed by 860 × ***g*** for 8 min at 4 °C. The resulting pellets were resuspended in homogenization buffer and lysed by passage through a 23‐gauge needle eight times. Lysates were centrifuged at 860 × ***g*** for 8 min at 4 °C to obtain post‐nuclear supernatants.

Membrane and cytosolic fractions were separated by ultracentrifugation of the post‐nuclear supernatants at 100 000 × ***g*** for 1 h at 4 °C. The membrane pellets were resuspended in RIPA buffer supplemented with a protease inhibitor cocktail prior to immunoblot analysis.

For preparation of total cell lysates, cells were washed three times with ice‐cold PBS and incubated on ice for 10 min in lysis buffer (50 mm Tris/HCl, pH 7.4, 150 mm NaCl, 1 mm EDTA, 1% Triton X‐100) supplemented with protease and phosphatase inhibitor cocktails. Cells were harvested by scraping and further lysed by passing the suspension through a 27‐gauge needle five times. Lysates were centrifuged at 13 000 × ***g*** for 5 min at 4 °C, and the supernatants were collected as soluble extracts.

### Western blotting

Protein samples were separated by SDS/PAGE and transferred onto PVDF membranes using a Trans‐Blot Turbo system (Bio‐Rad, Hercules, CA, USA). Membranes were blocked with 2% skim milk in TBS containing 0.1% Tween‐20 for 2 h at room temperature, followed by incubation with primary antibodies overnight at 4 °C. After washing, membranes were incubated with HRP‐conjugated secondary antibodies for 1 h at room temperature. Immunoreactive bands were visualized using an ECL Prime detection kit (GE Healthcare, Buckinghamshire, UK). Band intensities were quantified using ImageJ software, and 14‐3‐3ζ expression levels were normalized to β‐actin.

### Preparation of liposomes

Small unilamellar vesicles (SUV) of PS in Ham's F‐12 medium were prepared as described previously [9].

### Cell viability assay

Cells were seeded in 48‐well plates 1 day prior to the assay. PS liposomes were added to cells in antibiotic‐free growth medium (250 μL per well) and incubated for 24 h. Cell Count Reagent SF (25 μL per well) was added during the final 4 h of incubation.

Subsequently, 100 μL of the reaction mixture from each well was transferred to a 96‐well plate, and absorbance was measured at 490 nm with a reference wavelength of 630 nm using an iMark microplate reader (Bio‐Rad).

## Results

### The PS‐resistant CHO‐K1 cell mutant PSR406 is defective in flip‐flop‐dependent cytosolic incorporation of exogenous PS

We previously demonstrated that exogenously added excess phosphatidylserine (PS) is cytotoxic to CHO‐K1 cells, and we subsequently isolated a mutant cell line PSR406 (Phosphatidyl Serine Resistant 406) which is resistant to exogenous PS [5]. Compared to wild‐type CHO‐K1 cells, PSR406 exhibits a defect in endocytosis‐independent cytosolic incorporation of PS [5]. To further investigate this phenotype, CHO‐K1 and PSR406 were incubated with C_6_‐NBD‐PS at 6 °C for 5 min. Under these conditions, C_6_‐NBD‐PS labeled the cell surface of both cell types (Fig. 1A,D,G,J). After a subsequent incubation at 37 °C for 15 min, C_6_‐NBD‐PS was internalized in CHO‐K1 cells, resulting in diffuse fluorescence throughout the cytoplasm—presumably due to lipid diffusion within intracellular membranes following flip‐flop on the plasma membrane (Fig. 1B,H). In contrast, in PSR406 cells, the fluorescence remained localized to the plasma membrane (Fig. 1E,K). Notably, we did not observe any fluorescent endocytic vesicles in wild‐type cells under these experimental conditions (Fig. 1C,I). Removal of cell surface C_6_‐NBD‐PS by FCS confirmed that the lipid was not internalized to PSR406 cells (Fig. 1F,L). These findings suggest that flip‐flop is the major mechanism for the cytosolic incorporation of C_6_‐NBD‐PS in CHO‐K1 cells under these experimental conditions, and PSR406 is defective in this process.

**Fig. 1:**
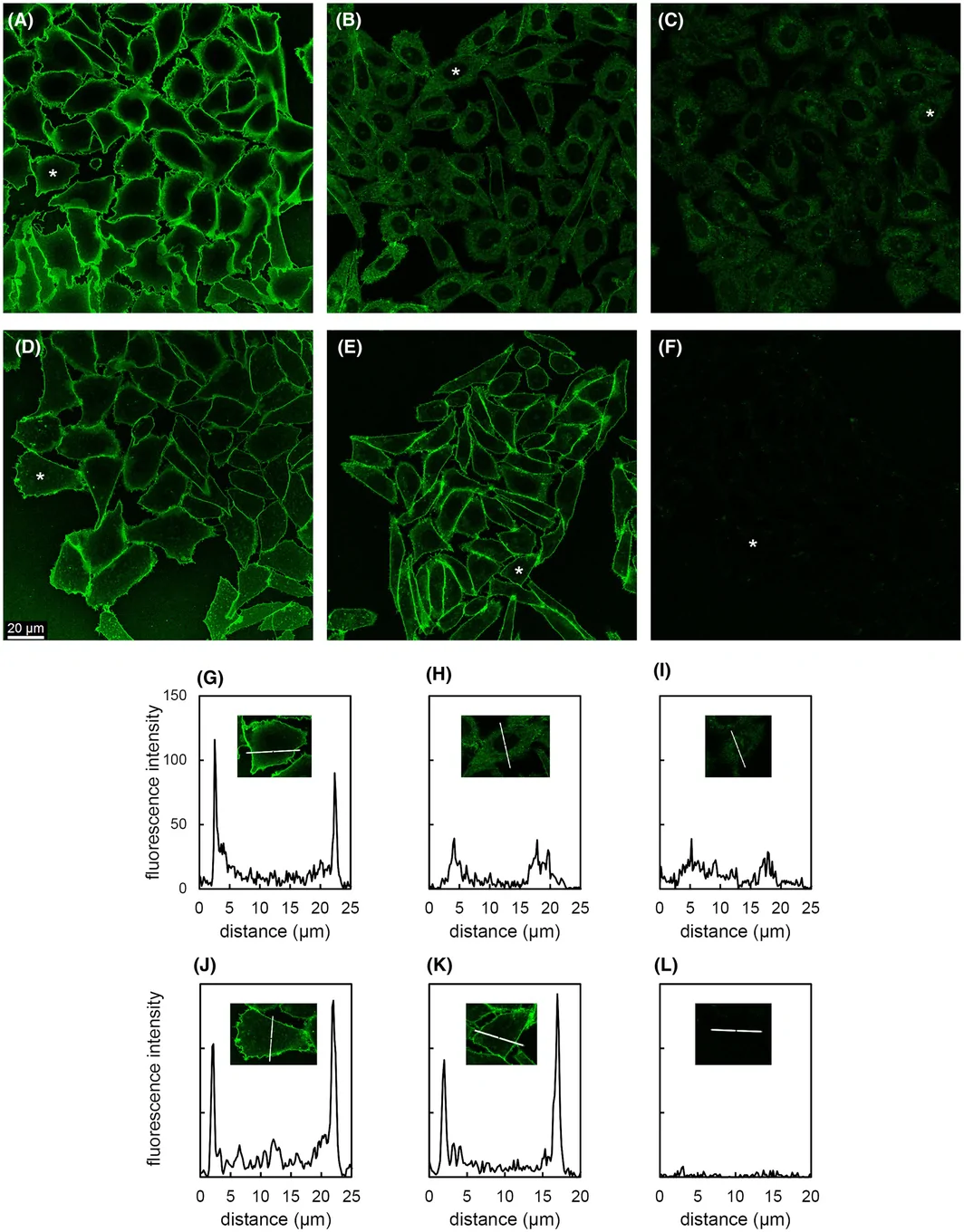
PSR406 is defective in cytosolic incorporation of a fluorescent PS analog. Wild‐type CHO‐K1 cells (A–C, G–I) and PSR406 mutant cells (D–F, J–L) were incubated with 3 μm C_6_‐NBD‐PS at 6 °C for 5 min (A, D, G, J). After washing with ice‐cold FCS‐free DMEM/Ham's F‐12 medium, cells were further incubated at 37°C for 15 min (B, E, H, K). To remove cell surface‐associated C_6_‐NBD‐PS, cells were subsequently incubated twice in Ham's F‐12 medium containing 10% FCS at 6 °C for 10 min (C, F, I, L). Images were acquired using a TCS SP8 confocal microscope, as described in Materials and methods. Scale bar, 20 μm. Cells indicated by an asterisk (*) in panels A‐F were subjected to fluorescence intensity analysis (G‐L). Graphs in panels G‐L show fluorescence intensity profiles along the white lines indicated in the corresponding image panels. Representative data from at least three experiments are shown.

To further define the mechanism of PS incorporation, cells were treated with dynasore, a GTPase inhibitor that rapidly and reversibly blocks dynamin activity [10]. In addition to inhibiting dynamin‐dependent endocytosis, dynasore has been reported to disrupt lipid raft organization in a dynamin‐independent manner [11, 12]. Thus, dynasore effectively suppresses both dynamin‐ and raft‐dependent endocytic pathways.

As shown in Fig. S1, dynasore treatment markedly inhibited the uptake of fluorescent dextran, confirming efficient inhibition of endocytosis under our experimental conditions. In contrast, cytosolic incorporation of C_6_‐NBD‐PS was not affected (Fig. 2). These findings indicate that C_6_‐NBD‐PS incorporation in CHO‐K1 cells occurs in an endocytosis‐independent manner.

**Fig. 2:**
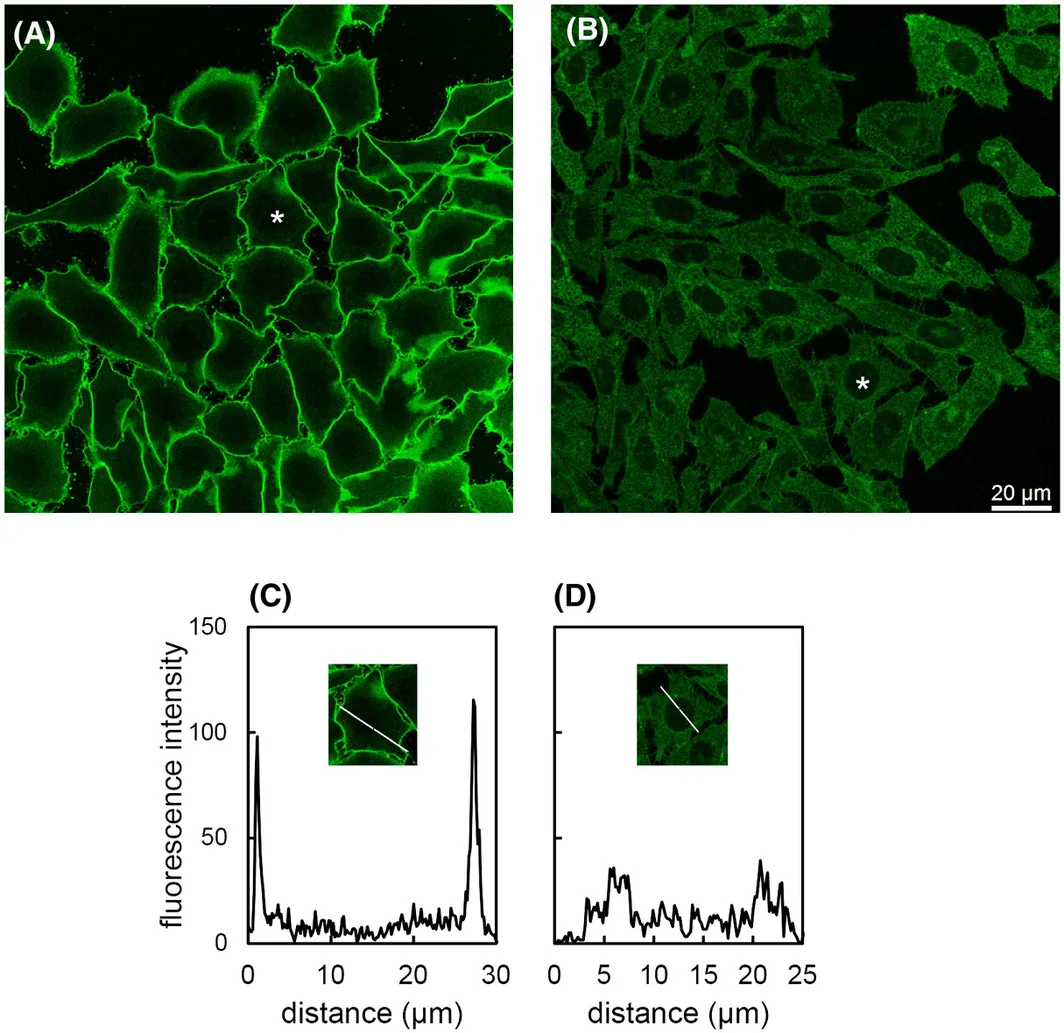
CHO‐K1 cells incorporate C_6_‐NBD‐PS in an endocytosis‐independent manner. CHO‐K1 cells were incubated with 3 μm C_6_‐NBD‐PS at 6 °C for 5 min (A, C). After washing, cells were further incubated at 37 °C for 15 min in the presence of 100 μm dynasore (B, D). Images were acquired using a TCS SP8 confocal microscope as described in Materials and methods. Scale bar, 20 μm. Cells indicated by an asterisk (*) in panels A and B were subjected to fluorescence intensity analysis (C, D). Graphs in panels C and D show fluorescence intensity profiles along the white lines indicated in the corresponding image panels. Representative results from at least three independent experiments are shown.

### 14‐3‐3ζ is deficient in PSR406

To identify proteins responsible for PS resistance and defect of flip‐flop of C_6_‐NBD‐PS in PSR406 cells, we performed mRNA subtraction cloning between CHO‐K1 and PSR406 cells. Initial screening revealed a dramatic decrease in 14‐3‐3ζ mRNA levels in PSR406. The 14‐3‐3 protein family comprises several isoforms, and isoforms ζ, β, γ, ε, η, θ and σ were expressed in CHO cells. We next quantitated the mRNA of each isoform by RT‐PCR (Fig. 3A). This analysis confirmed a significant reduction in the ζ isoform in PSR406. Interestingly, the γ isoform was upregulated by approximately 2.5‐fold in PSR406 compared to CHO‐K1 cells. Figure 3B compared the protein levels of 14‐3‐3ζ between CHO‐K1 and PSR406 cells. In CHO‐K1 cells, 14‐3‐3ζ was predominantly localized in the cytosolic fraction, with a smaller amount present in the membrane fraction. In contrast, in PSR406 cells, 14‐3‐3ζ levels were markedly reduced in both cytosolic and membrane fractions.

**Fig. 3:**
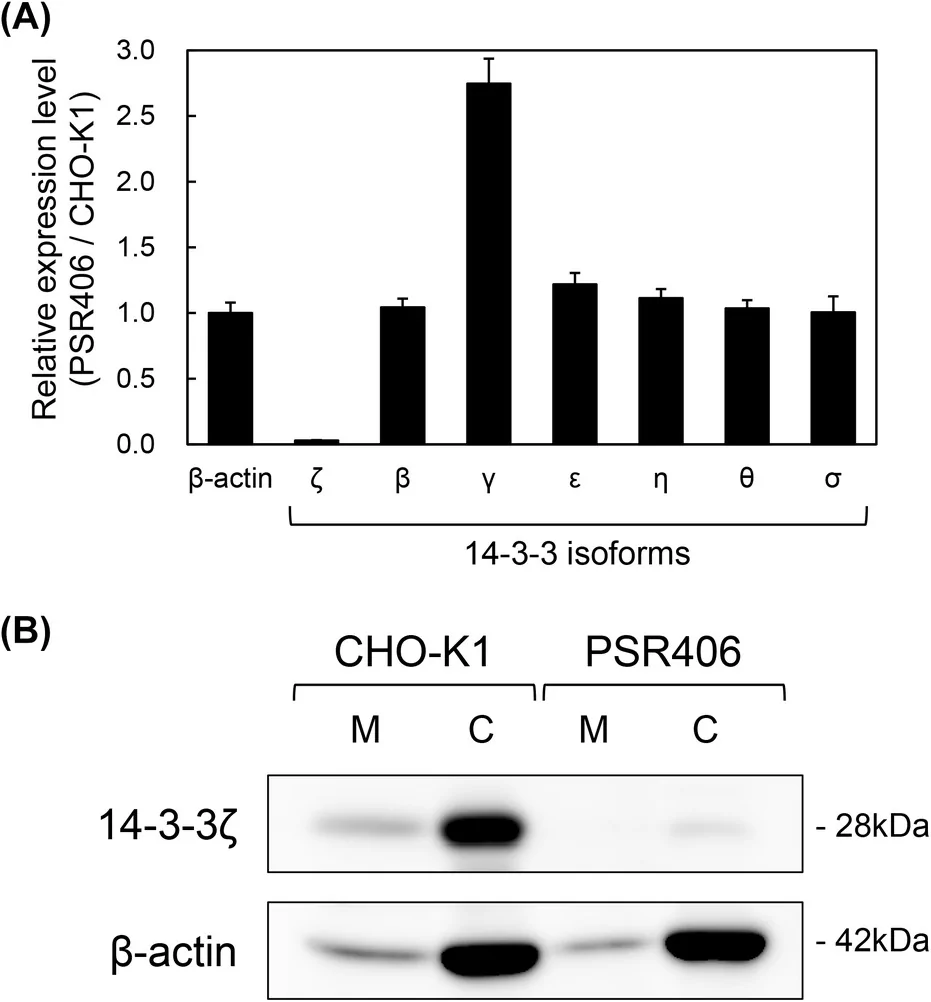
14‐3‐3ζ is deficient in PSR406 mutant cell. (A) mRNA levels of 14‐3‐3 isoforms in CHO‐K1 and PSR406 cells were analyzed by RT‐PCR. Expression ratios (PSR406/CHO‐K1) were normalized to β‐actin. Data represent the means ± SD of four experiments. (B) Endogenous 14‐3‐3ζ protein levels in membrane fraction (M) and cytosolic fractions (C) were analyzed by Western blotting.

### Knockdown of 14‐3‐3ζ confers resistant to exogenous PS and slows the flip‐flop of a fluorescent PS analog

These results suggest that the deficiency of 14‐3‐3ζ may contribute to PS resistance and the defect in flip‐flop of fluorescent PS analog in PSR406 cells. To test this hypothesis, we generated 14‐3‐3ζ knockdown cells using siRNA. As shown in Fig. 4A, 14‐3‐3ζ protein levels were significantly reduced following siRNA treatment. Knockdown cells exhibited partial resistance to exogenous PS (Fig. 4B). We next examined the cytosolic incorporation of C_6_‐NBD‐PS in knockdown cells (Fig. 5). After incubation with C_6_‐NBD‐PS at 7 °C for 5 min, both control siRNA (GFP) and 14‐3‐3ζ siRNA‐treated cells displayed similar plasma membrane labeling, comparable to wild‐type CHO‐K1 cells (Fig. 5A–C,G–I). Upon shifting the temperature to 37 °C, fluorescence was observed in the cytoplasm in wild type and control siRNA‐treated cells (Fig. 5D,E,J,K), indicating successful internalization of the lipid. In contrast, in 14‐3‐3ζ knockdown cells, fluorescence remained localized to the plasma membrane (Fig. 5F,L). No fluorescently labeled endocytic vesicles were observed in any of the three cell types under these conditions.

**Fig. 4:**
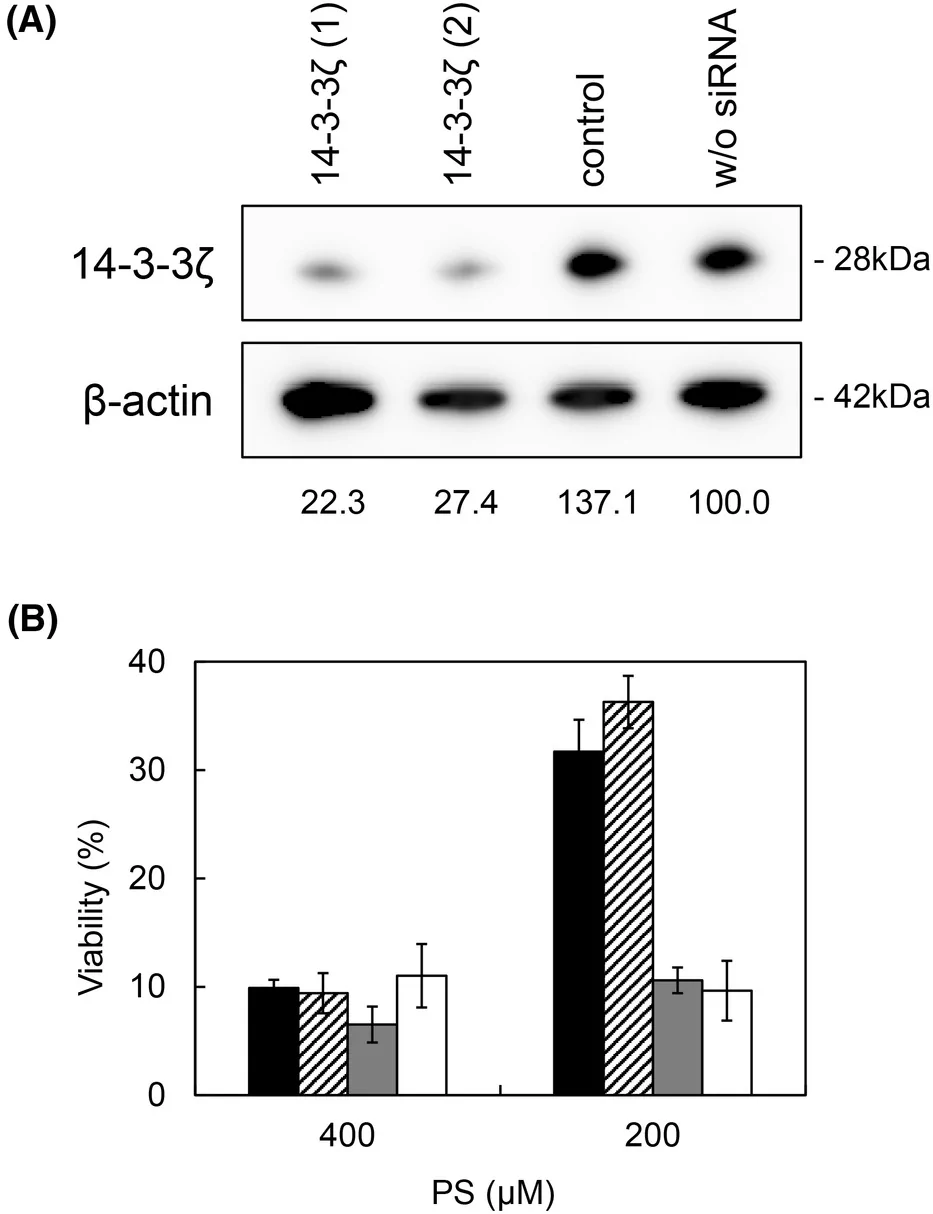
Knockdown of 14‐3‐3ζ confers partial resistance to exogenous PS. (A) Immunoblot analysis of 14‐3‐3ζ protein levels in CHO‐K1 cells treated with siRNAs targeting 14‐3‐3ζ (siRNA‐1 and siRNA‐2), a control siRNA (GFP), or left untreated. Relative expression levels (%) normalized to β‐actin are indicated below the blots. (B) CHO‐K1 cells treated with siRNA‐1 (black bars), siRNA‐2 (striped bars), control siRNA (GFP, gray bars), or untreated (white bars) were incubated with PS for 24 h. Cell viability was determined as described in Materials and Methods. Data represent the mean ± SD of four independent experiments.

**Fig. 5:**
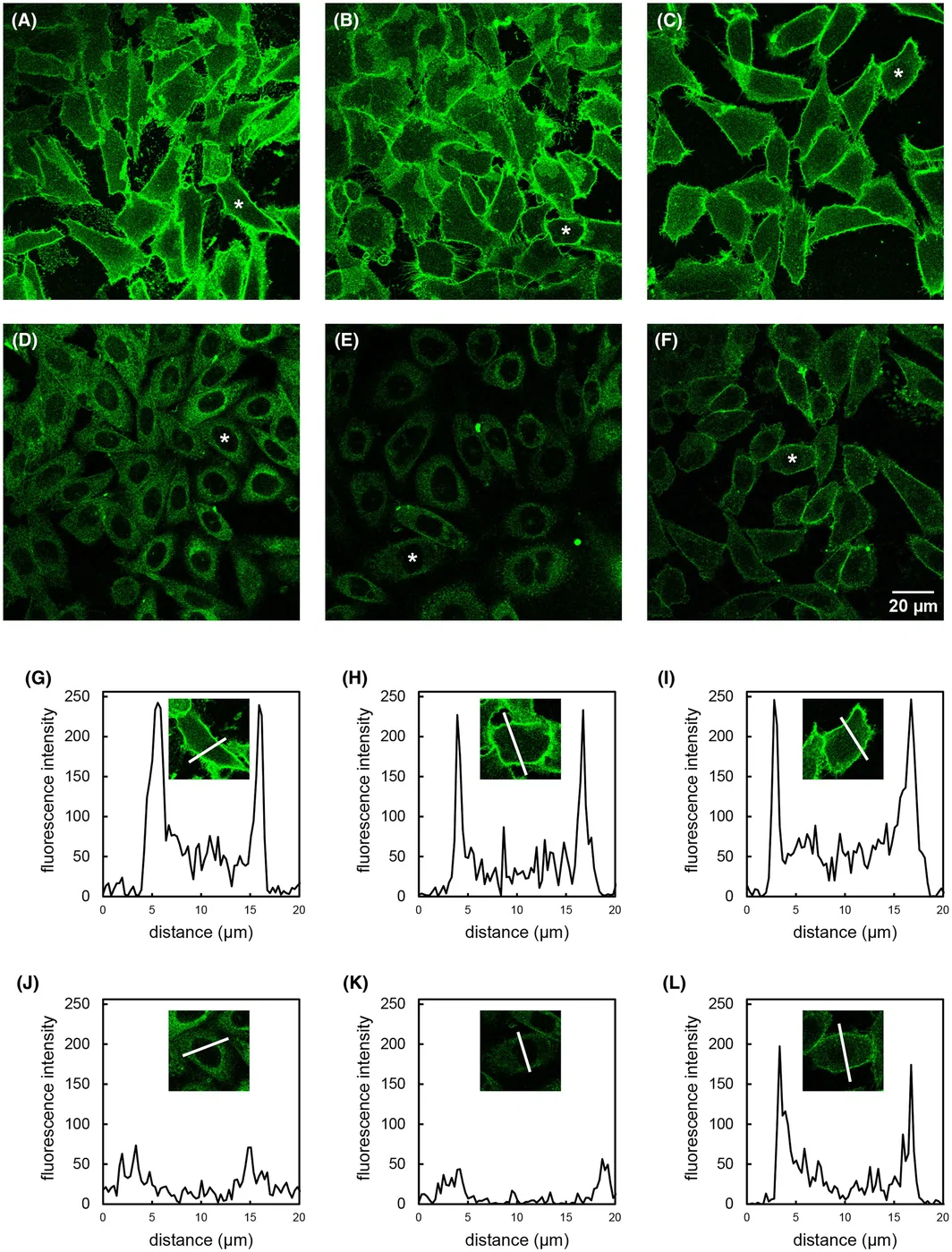
14‐3‐3ζ knockdown cells are defective in the cytosolic incorporation of fluorescent PS analog. Untreated CHO‐K1 cells (A, D, G, J), control siRNA‐treated cells (B, E, H, K), and 14‐3‐3ζ siRNA‐2‐treated cells (C, F, I, L) were incubated with 3 μM C_6_‐NBD‐PS at 7 °C for 5 min (A–C, G–I). After washing with ice‐cold FCS‐free DMEM/Ham's F‐12 medium, cells were further incubated at 37 °C for 15 min (D–F, J–L). Images were acquired using an LSM510 confocal microscope, as described in Materials and methods. Scale bar, 20 μm. Cells indicated by an asterisk (*) in panels A‐F were analyzed for fluorescence intensity along the white lines shown, and the corresponding intensity profiles are displayed in panels G–L. Representative images from at least three independent experiments are shown.

### Knockdown of 14‐3‐3ζ does not enhance PS resistance in ATP11C‐deficient cells

14‐3‐3ζ may interact with ATP11C directly or alternatively regulate PS internalization independently of ATP11C. 14‐3‐3 proteins are known to associate with a wide range of functionally diverse proteins, including kinases, phosphatases, and transmembrane receptors [13, 14, 15, 16]. In addition, interactions with negatively charged membrane phospholipids have been reported [17].

To determine whether 14‐3‐3ζ and ATP11C function synergistically in regulating PS sensitivity, we examined the effect of 14‐3‐3ζ knockdown in UPS‐1 cells harboring a nonsense mutation in ATP11C (Fig. 6). PS sensitivity was assessed following RNAi‐mediated depletion of 14‐3‐3ζ.

**Fig. 6:**
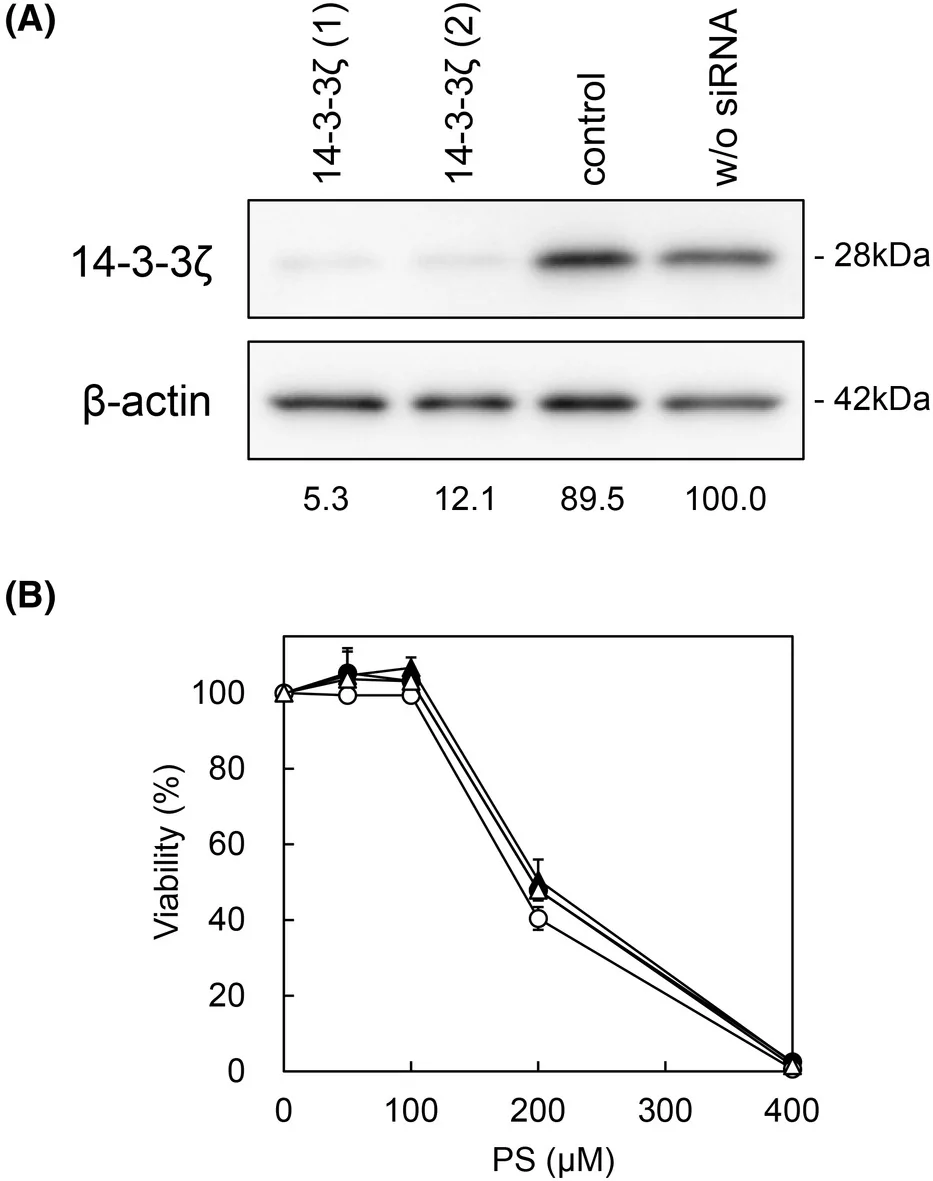
14‐3‐3ζ Knockdown does not affect PS sensitivity of UPS‐1 cells. (A) Immunoblot analysis of 14‐3‐3ζ protein levels in UPS‐1 cells treated with siRNAs targeting 14‐3‐3ζ (siRNA‐1 and siRNA‐2), a control siRNA (GFP), or left untreated. Relative expression levels (%) normalized to β‐actin are indicated below the blots. (B) UPS‐1 cells treated with siRNA‐1 (black circles), siRNA‐2 (black triangles), control siRNA (GFP, white circles), or untreated (white triangles) were incubated with PS for 24 h. Cell viability was determined as described in Materials and methods. Data represent the mean ± SD of four independent experiments.

No significant difference in PS sensitivity was observed between 14‐3‐3ζ depleted cells and control cells. These results indicate that loss of 14‐3‐3ζ does not further enhance PS resistance in ATP11C‐deficient cells, suggesting that 14‐3‐3ζ and ATP11C do not act synergistically in this context.

## Discussion

In this study, we identified 14‐3‐3ζ as a key factor responsible for resistance to exogenous PS and the endocytosis‐independent cytosolic incorporation of C_6_‐NBD‐PS in PSR406. Similar to PSR406, UPS‐1 cells isolated from Hanada and Pagano [3] also exhibits defect in endocytosis‐independent cytosolic incorporation of C_6_‐NBD‐PS. Recently ATP11C‐a member of the P4‐ATPase family‐was identified as the causative protein responsible for the defect in UPS‐1 cells [3, 4].

The 14‐3‐3 family of adaptor proteins plays diverse and essential roles in cellular regulation, including the control of cell proliferation, metabolism, adhesion, and apoptosis. These functions are mediated through interactions with a broad range of targets, such as enzymes, cytoskeletal components, signaling molecules, and tumor suppressors [13, 14, 15, 16]. In eukaryotic cells, 14‐3‐3 proteins are ubiquitously expressed, and in mammals, the isoforms ζ, β, γ, ε, η, θ, and σ have been identified. Each isoform exhibits a degree of binding specificity and associates with a distinct subset of the more than 500 known 14‐3‐3‐interacting proteins.

In PSR406 cells, we observed a marked reduction in the ζ isoform accompanied by an upregulation of the γ isoform. The PS‐resistant phenotype, together with the impaired flip‐flop of fluorescent PS analogues in 14‐3‐3ζ knockdown cells, indicates that the loss of the ζ isoform—rather than increased expression of the γ isoform—is responsible for the defect in transbilayer PS movement at the plasma membrane.

Interestingly, a recent study showed that 14‐3‐3ζ deficiency in platelets leads to PS exposure on the cell surface [18]. This phenotype was associated with sustained ATP levels and increased mitochondrial respiratory reserve, suggesting that ATP availability may regulate outward PS translocation. In contrast, our results indicate that 14‐3‐3ζ is required for inward PS transbilayer movement. Since ATP is known to enhance inward PS flipping via P4‐ATPases at the plasma membrane, it is likely that 14‐3‐3ζ regulates a different target or mechanism than that implicated in the outward movement observed in platelets.

Given the broad interaction profile of 14‐3‐3 proteins, it remains challenging to identify the direct molecular target responsible for the impaired PS incorporation observed in 14‐3‐3ζ‐deficient cells. Notably, 14‐3‐3ζ knockdown does not further enhance PS resistance in ATP11C‐deficient cells. These findings indicate that 14‐3‐3ζ plays a regulatory role in ATP11C‐dependent transbilayer movement of PS at the plasma membrane. Further studies will be necessary to delineate the precise molecular mechanisms involved.

In conclusion, together with the findings of Schoenwaelder et al. [18], our results suggest that 14‐3‐3ζ plays a dual role in regulating both inward and outward transbilayer movement of PS at the plasma membrane.

## Author contributions

Conceptualization: AY‐H, TK; Methodology: AY‐H, AH, TK; Investigation: AY‐H, AH; Visualization: AY‐H; Supervision: MT, TK; Writing‐original draft: AY‐H, TK; Writing‐review & editing: AY‐H, AH, MT, TK.

## Supporting information


**Fig. S1.** Dynasore inhibits fluorescent dextran endocytosis in CHO‐K1 cells.

## Data Availability

The data used and analyzed during the current study are available from the corresponding author upon reasonable request.
