# Supplementary material for: An isoform of 14‐3‐3 protein regulates transbilayer lipid movement at the plasma membrane
Source: FEBS Lett. 2026 Apr 15;600(20):3041–50. doi: 10.1002/1873-3468.70338 (PMC13703764; doi:10.1002/1873-3468.70338)
Supplement: Supplementary file 1 [file FEB2-600-3041-s001.pdf]

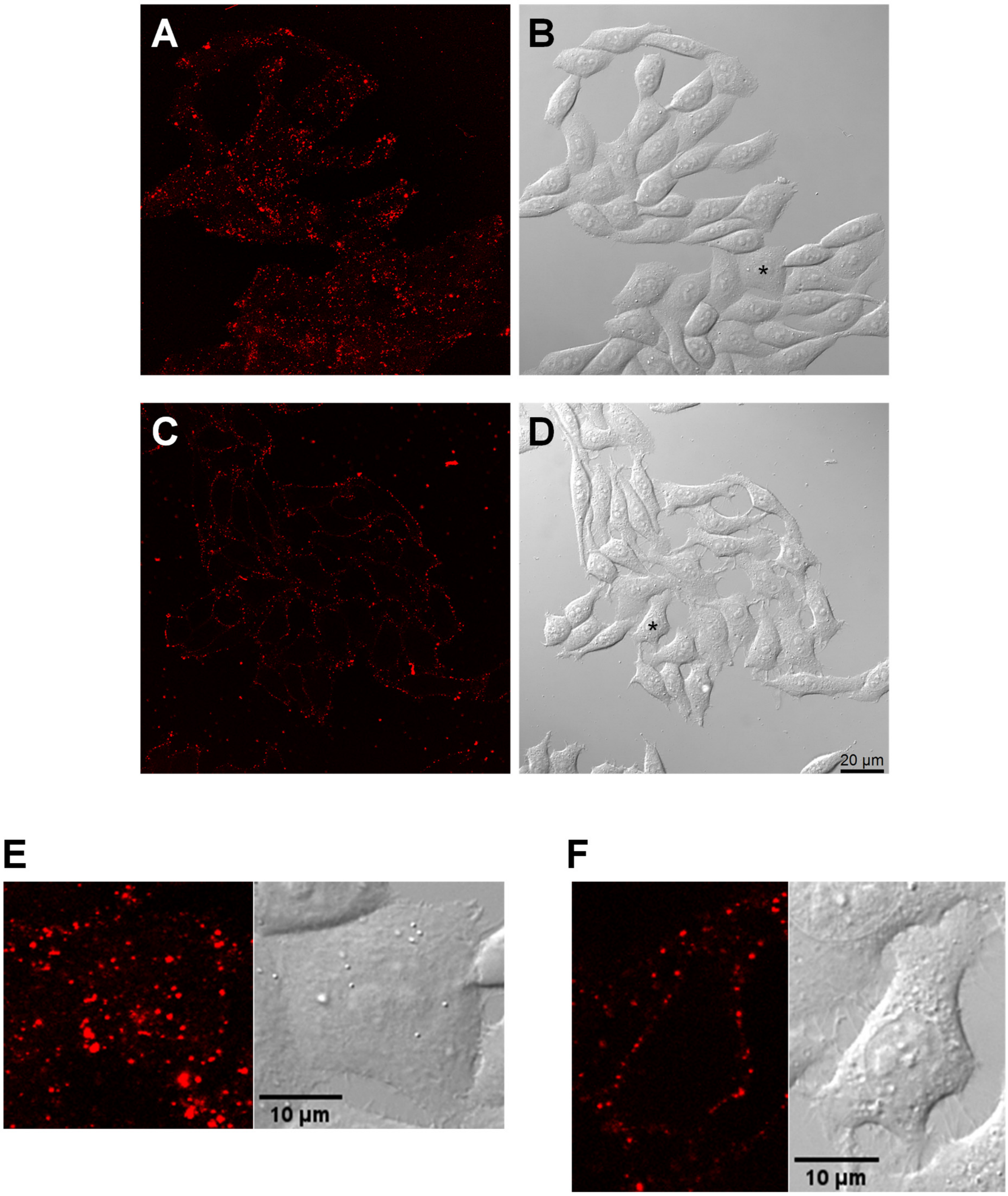

Supplementary Fig. S1. Dynasore inhibits fluorescent dextran endocytosis in CHO-K1 cells.
(A, B, E) CHO-K1 cells were incubated with 0.5 mg/ml Alexa Fluor™ 594–dextran at 37 °C for 15 min. **(C, D, F)** CHO-K1 cells were pretreated with 100 µM dynasore at 37 °C for 10 min and subsequently incubated with 0.5 mg/ml Alexa Fluor™ 594–dextran for 15 min at 37 °C in the continued presence of 100 µM dynasore. Images were acquired using a TCS SP8 confocal microscope as described in **Materials and methods**. Scale bar, 20 µm. **(E, F)** Higher-magnification images of the cells indicated by an asterisk (*) in panels **B** and **D**, respectively. Scale bar, 10 µm.
